# High-ranking alleviates male local competition in lek mating systems

**DOI:** 10.1038/s41598-018-33292-3

**Published:** 2018-10-12

**Authors:** Fabio Giavazzi, Nicola Saino, Alberto Vailati

**Affiliations:** 1 Dipartimento di Biotecnologie Mediche e Medicina Traslazionale, Università degli Studi di Milano, Milano, Italy; 2 Dipartimento di Scienze e Politiche Ambientali, Università degli Studi di Milano, Milano, Italy; 3 Dipartimento di Fisica, Università degli Studi di Milano, Milano, Italy

## Abstract

Territoriality entails demanding social interactions with competing individuals, typically males. Variation in quality of males can be predicted to affect the spatial arrangement of territories. We present a model aimed at understanding the spatial properties of territories on leks, where the presence of a hierarchy in a population of males leads to the clustering of individuals around high-ranking ‘hotshot’ males. The hierarchy results in a decrease in the number of nearest neighbors interacting directly with high-ranking males, with potential socio-sexual benefits for such males.

## Introduction

Lekking is a mating system that consists of aggregations of males into mating arenas (‘leks’) that females visit with the primary purpose of choosing one of the displaying males as a sexual mate. Lekking is widespread across animal taxa as diverse as insects and vertebrates, suggesting that it has appeared repeatedly during evolution^1^. Such widespread occurrence of lekking calls for the development of a general model based on a generic hierarchical interaction between individuals, with the aim of grabbing the essence of the formation of mating arenas determined by asymmetric competitive interactions between neighboring males in the presence of a hierarchy.

Most of the available models of territorial behavior assume that the population is made of identical individuals interacting symmetrically. At high density of individuals, territories may cover all the available space, potentially leading to complex social interactions among territory owners. Spatial models of territoriality predict that when space is saturated and the environment is homogeneous, territories take a polygonal shape determined by the interactions of any territory owner with its nearest neighbors, whose territory shares an edge with that of any focal individual^2–5^.

The partitioning of space arising from interactions between nearest neighbors can be conveniently expressed by a Voronoi diagram, where each territory comprises the points that are closer to the territory owner than to any other individual^6^. Hasegawa and Tanemura (HT) developed a geometrical model for the distribution of individuals exhibiting territorial behavior^3,4^. The HT model assumes that each individual adjusts its own position in the attempt of occupying the center of its territory. For individuals of equal ‘strength’ initially positioned at random, the spatiotemporal dynamics emerging from this behavioral rule leads to a spatial arrangement of territories where the mean and modal value of the frequency distribution of the number of nearest neighbors is 6 and the modal size of territories is equal to the mean space available per individual. The predictions from this model have been confirmed by observations on fish and birds^3,7^.

The spatial distribution of individuals is expected to be dramatically altered by the presence of a socio-sexual hierarchy among population members^8^, a remarkable real example being lekking species. Once on the lek, males compete for copulating with females prospecting for mating partners, by displaying ornaments such as feathers or antlers, scent, by fighting or by vocalizing, depending on the species. The frequency distribution of copulations is highly skewed among males, with high-ranking ones monopolizing most of the females and copulations. On leks, females cannot typically be coerced to copulate, and the frequency of copulations therefore represents a reliable clue to gauge the rank of individual males inside the lek as determined by female mate preference.

In this work we present a model for the formation of leks in a territorial population of animals. Several models have been proposed for the formation of leks^1^. Here we focus on a “hotshot” model^9,10^, which posits that females prefer attractive, “hotshot” males as mates and therefore tend to cluster around them on leks. According to this model, less attractive males are in turn also assumed to gather around the hotshot males during lekking behavior because this will boost their own chances of copulating with the females. This assumption is realistic because by gathering around the hosthots, non-preferred males will increase the chances of intercepting the females on the lek. For example, in natterjack toads (*Bufo calamita*) and treefrogs (e.g. *Hyla cinerea*) satellite males sit close to large, preferred males to gain access to copulations by sneaking^1^. Thus, in hotshot lekking systems, non-preferred males are apparently trading the costs of competition with superior males against the benefits of increasing their chances of intercepting females visiting the lek.

## Results

In our simple hierarchical model, the population is divided into two classes of males: high-ranking (‘dominant’) males and low-ranking (‘subordinate’). In a real population of lekking males, the rank of an individual is reflected by the number of copulations achieved by it with respect to the entire population. For example, field studies on lekking sage grouses, the highest-ranking male was responsible for about 50% of 105 copulations, three males of intermediate rank for most of the remaining 50%, while the 10 remaining individuals engaged either in a marginal number of copulations or in no copulations at all^11^. Another meaningful example is represented by lekking in the lance-tailed manakin (*Chiroxiphia lanceolata*)^12^. The males of this species can be divided into two main classes: a small class of males (22% of the population of males or less) with non-zero reproductive success and a large class of males (78% of the population of males or more) with zero reproductive success. Therefore, although the assumption of the presence of two main classes of ranks in our model may appear simplistic, it allows to catch the fundamental difference between a class of hotshot males achieving reproductive success and the rest of lekking males. Moreover, from the conceptual point of view, a two-ranks model allows to minimize the number of arbitrary parameters and to achieve a complete mapping of the parameter space. In our model, the rank is expressed by attributing to each individual in the high-rank class a weight w_HR_, and to those in the low-rank class a weight w_LR_ < w_HR_. The spatiotemporal dynamics of the population is entirely governed by two parameters: the fraction ϕ of low-ranking males with respect to the entire population, and the relative weight ρ = w_LR_/w_HR_ of low-ranking males with respect to the high-ranking ones.

**Figure 1:**
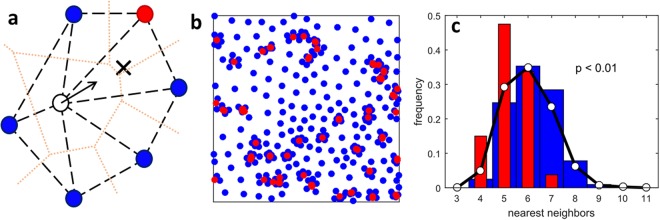
Hierarchical interaction between individuals with a weight ratio ρ = 0.2. (**a**) An individual (open circle) surrounded by high-ranking (red) and low-ranking (blue) nearest neighboring males moves towards their center of mass (cross). Here the weight of the high ranking male is 5 times larger than that of low ranking ones. The dotted line identifies the edges of territories defined as the region of space closer to the territory owner than to any other individual. Nearest neighbor individuals are those that share a boundary of their territories. (**b**) Stable spatial configuration of high-ranking (red) and low-ranking (blue) males obtained for ϕ = 0.8 and ρ = 0.2. In the initial configuration, individuals were randomly distributed. The final stable configuration shows the formation of clusters of low-rank individuals around high-rank ones. Each of these clusters represents a lek. (**c**) Histograms of the distribution of the number of nearest neighbors for high-ranking males (red), low-ranking males (blue), and the overall population (white dots); the histograms correspond to the configuration shown in panel b.

The population dynamics is determined by a behavioral rule similar to that adopted by Hasegawa and Tanemura^3^: at each time step each individual moves towards the center of mass of its nearest neighbors. In a condition of equal ranks (ρ = 1) each individual tends to occupy the center of its territory. This gives rise to a deterministic dynamics of the spatial distribution of individuals, which rapidly converges to a stable spatial configuration where the position of each individual is fixed (Supplementary Movie 1). In our model, the position of nearest neighbor individuals is weighted by their rank. This choice favors the proximity of individuals to high-ranking males (Fig. 1a). In the case of intermediate and large values of ρ, this rule eventually leads to a stable configuration where each individual sits in the weighted center of mass of its nearest neighbors (Fig. 1b, Supplementary Movie 2). In the case of small values of ρ ≈ 0.1, the dynamics leading to a stable configuration is strongly intermittent and accompanied by large fluctuations (Supplementary Movie 3). The time needed to reach a stable configuration increases gradually when the ratio ρ decreases, spanning more than four orders of magnitude (Supplementary Fig. 1).

**Figure 2:**
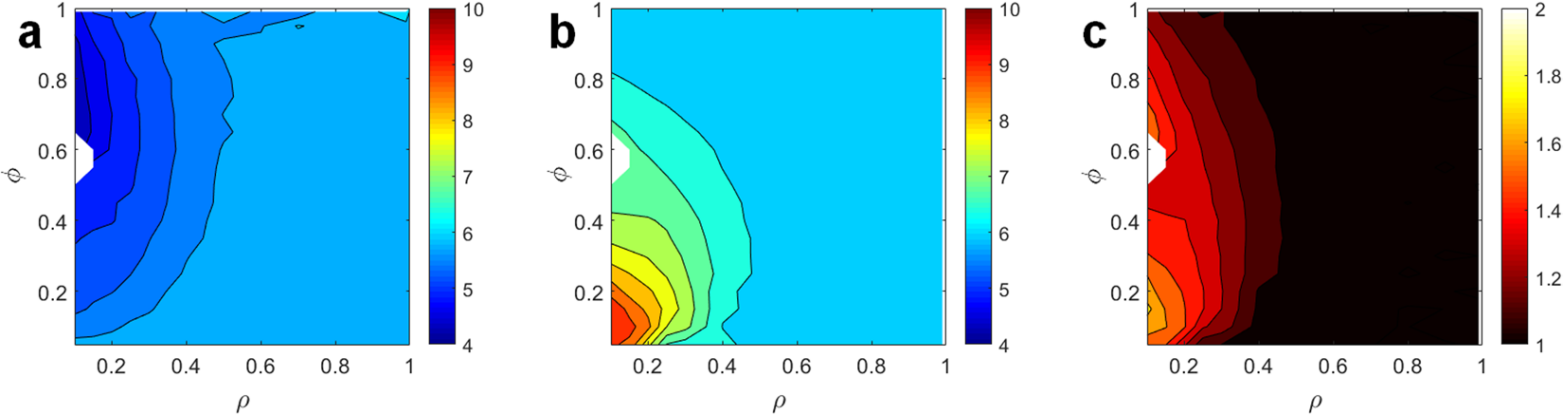
Mean number of nearest neighbors. (**a**) Mean number of nearest neighbors for high-ranking males, and (**b**) for low-ranking males. (**c**) ratio between the mean number of nearest neighbors for low-ranking relative to high-ranking males. All quantities are plotted as a function of the fraction ϕ of low-ranking males and the ratio ρ between the weights. The white regions correspond to combinations of parameters where a stable configuration was not attained after 4 × 10^6^ simulation steps.

An emerging feature of the final stable configuration is the variation in the number of nearest neighbors according to male rank (Fig. 1c): high-ranking males are surrounded on average by less than 6 nearest neighbors (Fig. 2a), with a minimum of 4 males at ρ ≈ 0.1, whereas low-ranking males are surrounded by more than 6 nearest neighbors, with a maximum of 9–10 at ρ ≈ 0.1 (Fig. 2b). The overall average number of nearest neighbors <n> for the entire population is invariably equal to 6 for all values of ρ and ϕ.

## Discussion

The observation of a *mean* of 6 nearest neighbors has been frequently advocated as a demonstration of a regular arrangement of individuals in space^2,13–15^. It should be rather noted that an average of 6 nearest neighbors is a geometrical property arising from Euler’s equation for a two dimensional network, which imposes that <n> must be equal to 6 for the entire population under the rather generic condition that a boundary point is shared at most by three territories. This condition is satisfied even in the case of territories generated by randomly distributed individuals^16,17^. As a consequence of Euler’s equation, the average number <n_HR_> and <n_LR_> of nearest neighbors for high-ranking and low-ranking males must obey the linear relation (1 − ϕ) <n_HR_> + ϕ <n_LR_> = 6. The additional advantage for high-ranking males can be expressed as the ratio <n_LR_>/<n_HR_> = 1 − 1/ϕ (6/<n_HR_> −1). In the case of individuals of equal weight, this ratio is equal to 1, while in the presence of variation in weight it falls in the range 1–1.7 (Fig. 2c), with largest advantages for high-ranking individuals arising at small values of ρ. Hence, at equilibrium high-ranking males have fewer nearest neighboring males to interact with compared to low-ranking males, despite the population-level average number of nearest neighbors is bound to be 6. High-ranking males may therefore accrue additional advantages over low-ranking males besides the fact that they are, by definition, attractive to females. These advantages may be mediated by shorter time spent in territorial interactions with neighboring males, and thus with more time available to sexual displays. In addition, females may credit males with fewer nearest neighbors as being of high quality for the mere fact that they have fewer nearest neighbors than average males. Our model leads to predict that for ρ = 0.1 the number of nearest neighbors ranges between 4 for high-ranking males (Fig. 2a) and 7–9 for low-ranking ones (Fig. 2b). These additional advantages may not be trivial.

**Figure 3:**
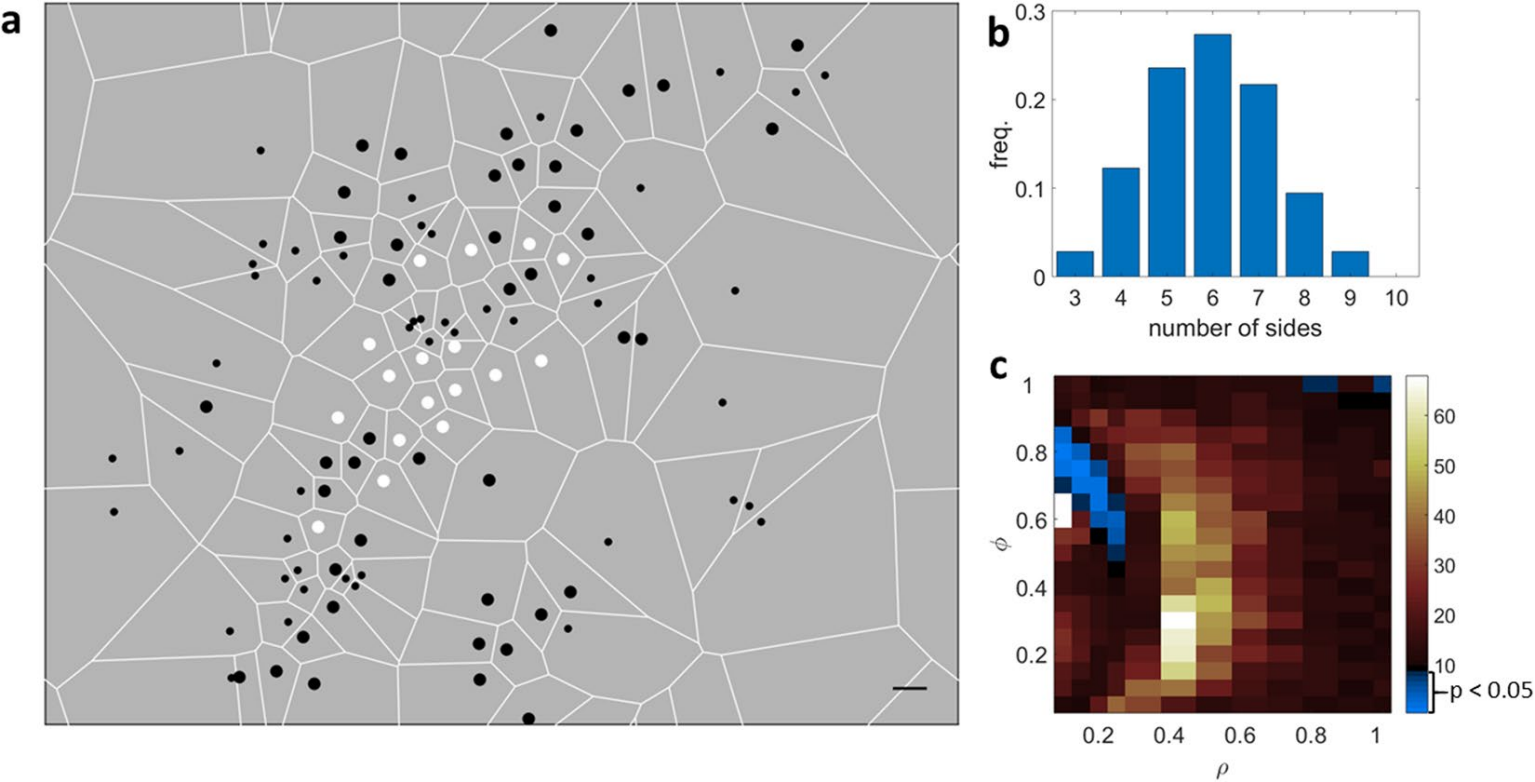
Comparison between the distribution of the number of nearest neighbors in a real population of MacQueens Bustard and in our model. (**a**) Spatial distribution of the display sites in the exploded lek of a population of MacQueen’s Bustard^19^. White circles represent radio-tagged individuals, black circles non-tagged individuals and dots undetermined observations. White lines delimit the Voroni cells generated by the population. Scale bar: 1 km. (**b**) Frequency distribution of the number of nearest neighbors for the spatial distribution shown in a, calculated as the number of sides of the associated Voronoi cells. (**c**) χ^2^ test-statistic calculated from the deviations between the observed frequency distribution shown in b and those expected from our model. The region where the null hypothesis can be accepted (assuming a significance level of 0.05) is highlighted in blue. The white regions correspond to combinations of parameters where a stable configuration was not attained after 4 × 10^6^ simulation steps.

The weight unbalance among individuals in real populations is difficult to estimate. As far as this can be gauged from the variance in copulation numbers, it can be large, as suggested by the fact that in some leks the few, most successful males monopolize a very large fraction of the copulations^1,11,12^. A test of the biological realism of our findings can be achieved by comparing the distribution of the number of nearest neighbors determined from field observation of a lekking population with those obtained from our model. This is a hard feat, because collecting data on large leks is technically difficult^18^. Recent studies of the spatial organization of MacQueen’s Bustard (*Chlamydotis macqueenii*) have shown that individuals organize into ‘exploded’ leks where the typical distance between individual display sites becomes of the order of 1 km and males tend to hold fixed territories^19^. This feature allows a reliable determination of the spatial distribution of display sites by using GPS and radio-tracking in combination with direct observation (Fig. 3a), and thus of the frequency distribution of the number of nearest neighbors (Fig. 3b).

To test the compatibility between the experimental distribution of nearest neighbors and that predicted by our model, we calculated the χ^2^ test-statistic and performed a Pearson’s χ^2^ test across all the parameters space (Fig. 3c). Assuming a significance level of 0.05, the critical value of χ^2^ is 9.5. The region of the parameter space where χ^2^ <9.5 and the null hypothesis can be accepted is highlighted in blue in Fig. 3c. As it can be appreciated from the figure, the compatibility region is rather narrow and corresponds to a region of the parameter space of our model were ϕ ≈ 0.8 and ρ ≈ 0.1, a configuration where low-ranking males are clustered around high-ranking ones, which benefit from a significant reduction of the number of nearest neighbors. Therefore, although the overall distribution of individuals in Fig. 3a appears to be compatible with a random distribution, it is also compatible with a hierarchical distribution, which becomes apparent only when a rank can be attributed to each individual. For this reason, a more stringent test would be represented by a ranking-resolved comparison between our model and the results of field studies, where the geometrical distributions for high-ranking and low-ranking individuals are considered separately. Unfortunately, such a test cannot be performed in the case of MacQueen’s Bustard, because the presented data are not ranking-resolved^19^.

In conclusion, the results of our model show that the presence of a hierarchy in a population of territorial animals gives rise to the development of a pyramidal system of interactions between individuals, where the number of nearest neighbors directly reflects the rank of an individual.

## Methods

Simulations were performed on populations of N =400 individuals hosted in a square box of side 1 with periodic boundary conditions. Each individual was initially attributed a random position. A number N_LR_ = ϕ N of individuals chosen at random in the population were attributed a low-rank weight w_LR_, while all the others a high-rank weight w_HR_. At each time step the positions **x**_i_ of the nearest neighbors of each individual were identified. The identification of nearest neighbors was performed through the Voronoi-Delauney tessellation algorithm: the space is divided into polygonal territories identified as the regions of space closer to an individual than to any other individual and nearest neighbor individuals are defined as those sharing part of the boundary of their territories. The weighted center of mass of nearest neighbors **x**_CM_=  Σ w_i_ **x**_i_/Σ w_i_ was calculated for each territory owner, which was moved at the midpoint of the segment connecting its initial position to the center of mass of its nearest neighbors. The reach of a stable configuration was determined by processing the mean square displacement MSD of individuals at each time step. When the MSD fell below a threshold value of 10^−14^ of the box size the final configuration was recorded and the simulation was stopped. Simulations not converging after 4 million steps were stopped and not included in the analysis (white regions of the diagrams in Figs 2 and 3c). Final configurations were determined systematically for different realizations of the system by varying the parameters ρ and ϕ in the range 0.1–1 with step 0.05. Ten independent realizations for each parameter set were processed to achieve a good statistical characterization of the system. The size of the statistical sample was dictated by the constraints imposed by processing time, which became prohibitively long for small values of ρ. Stable configurations obtained at the end of each simulation run were processed to determine the distribution of number of nearest neighbors for high-ranking and low-ranking individuals and for the entire population. To check the robustness of the two-rank model we implemented a similar model based on a larger number of discrete ranks. We found that the time needed to reach a stable configuration during the evolution of the system is determined by the ratio between the weights of lowest-rank males and highest-rank ones. However, the development of a model with a wide number of ranks requires to introduce a large number of arbitrary parameters, and results in the development of a “simulation”^2^ able to describe accurately the features of a specific system, rather than in the development of a general model able to grab the essential features of the lekking behavior in a wide range of animal species. For each combination of the parameters (ϕ, ρ), a difference in the distributions of the number of nearest neighbors for high-ranking and low-ranking males was tested by Mann-Whitney U-test. The α-level of the test was set at p < 0.05.

We compared the results of our simulations with the observations reported by Riou and Combreau on MacQueen’s Bustard^19^. We used the Voronoi-Delaunay tessellation algorithm to determine the distribution *T*(*n*) of the number of nearest neighbours for each of the ${N}_{0}=106$ display sites shown in Fig. 3a, by including tagged, non-tagged and undetermined individuals. To avoid a bias in the distribution determined by the absence of incomplete Voronoi cells related to display sites near the boundaries of the observation region, we added periodic boundary conditions to the pattern shown in Fig. 3a. This resulted in a mean number of nearest neighbors for the population <n>= 5.93 This value is compatible with the one expected for a planar tessellation with trivalent vertices, which, as discussed above, is constrained to 6^16,17^.

In order to check the compatibility between $T(n)$ and the distribution $F(n)$ of the number of sides obtained from our simulations, we performed a Pearson’s χ^2^ test for each combination of the parameters (ϕ, ρ). The p-value is calculated by comparing the value of the test-statistic ${\chi }^{2}={N}_{0}\{\frac{{[{\sum }_{n{ < }5}(F(n)-T(n))]}^{2}}{{\sum }_{n < 5}F(n)}+\frac{{[F(5)-T(5)]}^{2}}{F(5)}$ $+\frac{{[F(6)-T(6)]}^{2}}{F(6)}+\frac{{[F(7)-T(7)]}^{2}}{F(7)}+\frac{{[{\sum }_{n > 7}(F(n)-T(n))]}^{2}}{{\sum }_{n > 7}F(n)}\}$ to a chi-squared distribution with 4 degrees of freedom. The α-level of the test was set at p < 0.05.

### Code availability

The custom computer code used to generate results is available from the authors upon request.

## Electronic supplementary material

Supplementary Movie 1

Supplementary Movie 2

Supplementary Movie 3

Supplementary Information

## Data Availability

All data are available upon request.
