# Supplementary material for: High-ranking alleviates male local competition in lek mating systems
Source: Sci Rep. 2018 Oct 12;8:15189. doi: 10.1038/s41598-018-33292-3 (PMC6185937; doi:10.1038/s41598-018-33292-3)
Supplement: Supplementary file 4 — Supplementary Information [file 41598_2018_33292_MOESM4_ESM.pdf]

Supplementary information

High-ranking alleviates male local competition in lek mating systems

Fabio Giavazzi[1], Nicola Saino[2] and Alberto Vailati[3*]

1 Dipartimento di Biotecnologie Mediche e Medicina Traslazionale, Università degli Studi di Milano, Milano, Italy

2 Dipartimento di Scienze e Politiche Ambientali, Università degli Studi di Milano, Milano, Italy

3 Dipartimento di Fisica, Università degli Studi di Milano, Milano, Italy

* email: alberto.vailati@unimi.it

Contents:

1. Supplementary Fig. 1. Number of simulation steps required to reach a stable configuration.
2. Supplementary Fig. 2. Frequency distribution of the number of nearest neighbors.
3. Supplementary Movie 1. Spatio-temporal evolution of the system for ϕ=0.8 and ρ = 0.9. Red dots represent high-ranking males and blue dots low ranking males.
4. Supplementary Movie 2. Spatio-temporal evolution of the system for ϕ=0.8 and ρ = 0.2. Red dots represent high-ranking males and blue dots low ranking males.
5. Supplementary Movie 3. Spatio-temporal evolution of the system for ϕ=0.6 and ρ = 0.1. Red dots represent high-ranking males and blue dots low ranking males.

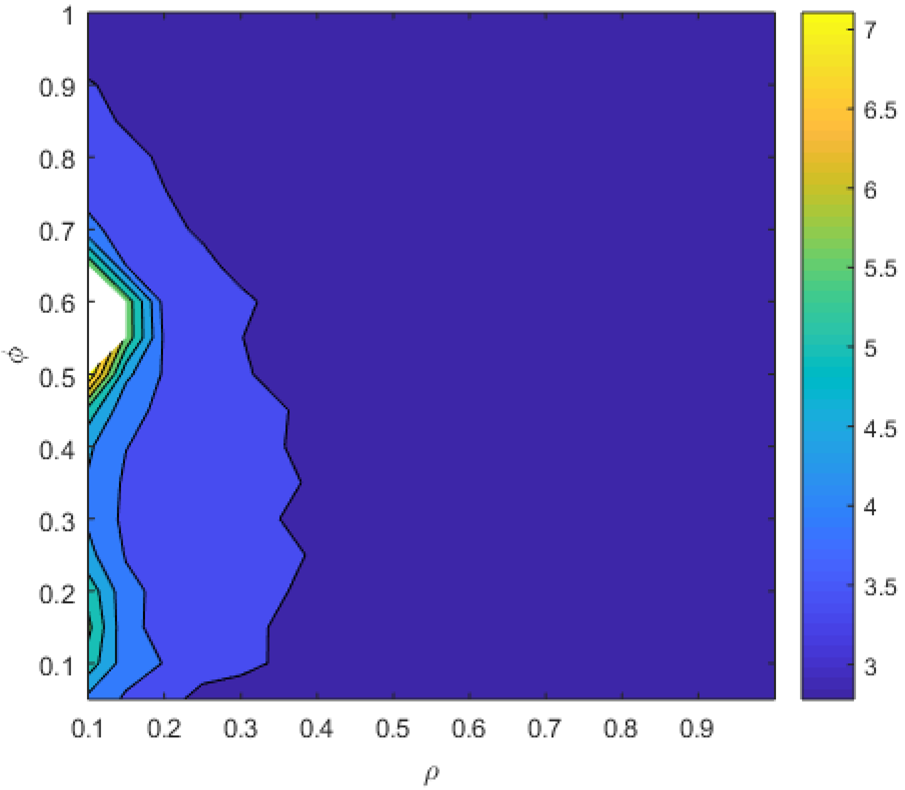

Supplementary Fig. 1. Number of simulation steps required to reach a stable configuration. The stable configuration corresponds to the condition where the mean square displacement of individuals during one simulation step fell below $1x10^{-14}$. Data are plotted on a Log10 scale.

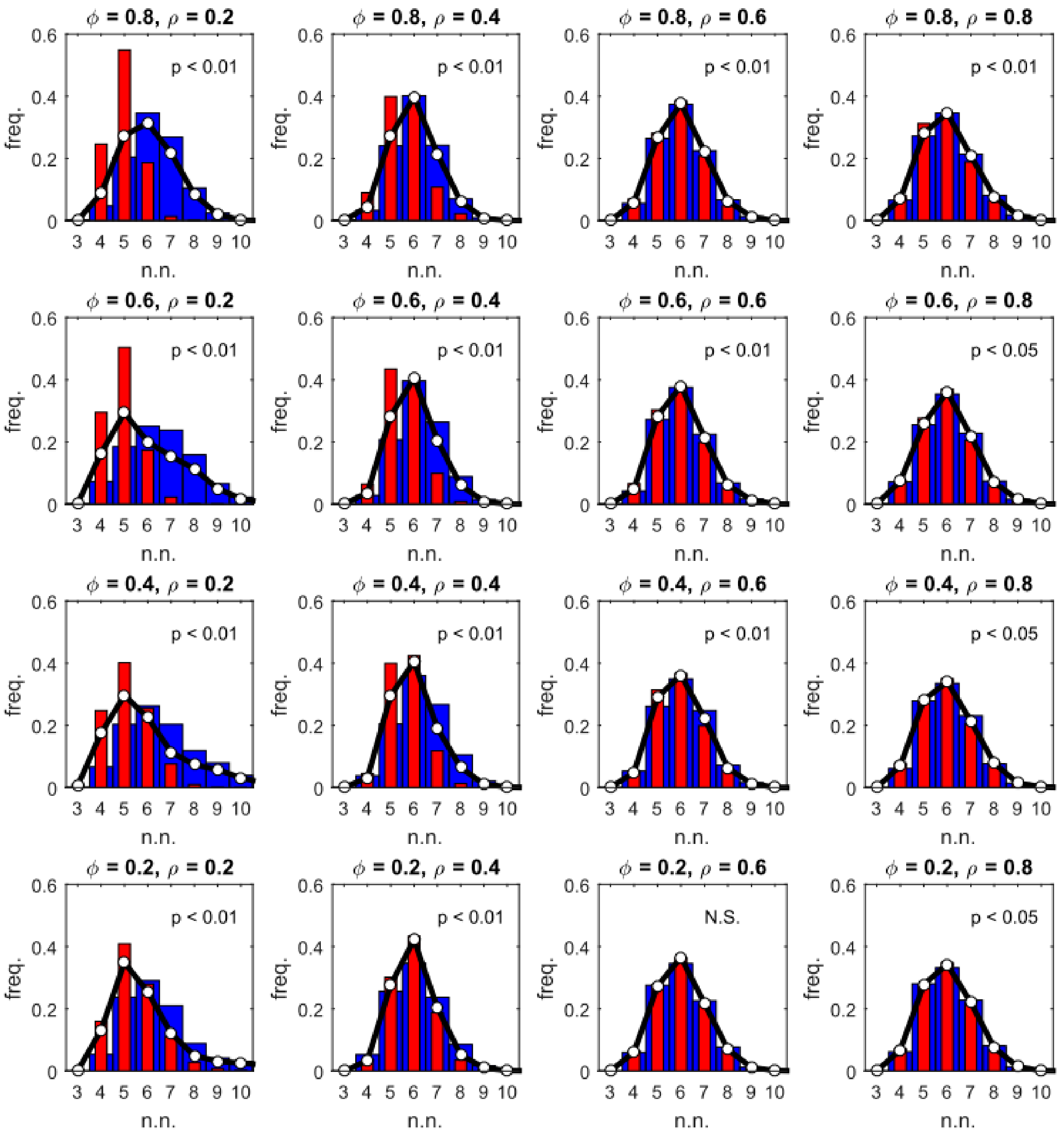

Supplementary Fig. 2. Frequency distribution of the number of nearest neighbors. Each panel corresponds to a different combination of the fraction ϕ of low-ranking males and of the ratio ρ between the weights of low- and high-ranking males. The frequency (freq.) distribution is obtained over 10 independent realizations, for the number of nearest neighbors (n.n.) of high-ranking males (red), low-ranking males (blue), and the overall population (white dots). The significance level of the p-value was determined with a Mann-Whitney U test on the distributions of high- and low-ranking males (N.S. = Not Significant).
